# Shared decision making, physicians’ explanations, and treatment satisfaction: a cross-sectional survey of prostate cancer patients

**DOI:** 10.1186/s12911-020-01355-z

**Published:** 2020-12-14

**Authors:** Kazuhiro Nakayama, Wakako Osaka, Nobuaki Matsubara, Tsutomu Takeuchi, Mayumi Toyoda, Noriyuki Ohtake, Hiroji Uemura

**Affiliations:** 1 Graduate School of Nursing Science, St. Luke’s International University, 10-1 Akashi-cho, Chuo-ku, Tokyo, 104-0044 Japan; 2 Faculty of Nursing and Medical Care, Keio University, Tokyo, Japan; 3 The Jikei University School of Medicine, School of Nursing, Tokyo, Japan; 4 Department of Breast and Medical Oncology, National Cancer Center Hospital East, Kashiwa, Japan; 5 NPO Prostate Cancer Patients Association, Hyogo, Japan; 6 Janssen Pharmaceutical K.K, Tokyo, Japan; 7 Department of Urology and Renal Transplantation, Yokohama City University Medical Center, Yokohama, Japan

**Keywords:** Prostate cancer, Hormonal therapy, Shared decision making, SDM-Q-9, SDM-Q-Doc, Effective decision making, Treatment satisfaction, Patients and healthcare providers communication, Information provision, Survey

## Abstract

**Background:**

Hormone therapy is one option for some types of prostate cancer. Shared decision making (SDM) is important in the decision making process, but SDM between prostate cancer patients receiving hormone therapy and physicians is not fully understood. This study tested hypotheses: “Patients’ perception of SDM is associated with treatment satisfaction, mediated by satisfaction with physicians’ explanations and perceived effective decision making” and “The amount of information provided to patients by physicians on diseases and treatment is associated with treatment satisfaction mediated by patients’ perceived SDM and satisfaction with physicians’ explanations.”

**Methods:**

This cross-sectional study was conducted using an online panel via a private research company in Japan. The participants in this study were patients registered with the panel who had received or were currently receiving hormone therapy for prostate cancer and physicians registered with the panel who were treating patients with prostate cancer. Measures used in this study included a nine-item Shared Decision Making Questionnaire, levels of satisfaction with physicians’ explanations and treatment satisfaction, and effective decision making for patients (feeling the choice is informed, value-based, likely to be implemented and expressing satisfaction with the choice), and a Shared Decision Making Questionnaire for Doctors. The hypotheses were examined using path analysis.

**Results:**

In total, 124 patients and 150 physicians were included in the analyses. In keeping with our hypotheses, perceived SDM significantly correlated with the physicians’ explanations and perceived effective decision making for patients, and satisfaction with physicians’ explanations and perceived effective decision making for patients were both related to treatment satisfaction. Although the amount of information provided to patients was correlated with the perceived SDM, it was indirectly related to their satisfaction with physicians’ explanations.

**Conclusions:**

When physicians encourage patients to be actively involved in making decisions about treatment through the SDM process while presenting a wide range of information at the start of hormone therapy, patients’ effective decision making and physicians’ explanations may be improved; consequently, the patients’ overall treatment satisfaction may be improved. Physicians who treat patients with prostate cancer may have underestimated the importance of SDM before starting hormone therapy, even greater extent than patients.

## Background

Shared Decision Making (SDM) is a “process in which patients, their families, and healthcare professionals share treatment options, benefits and harms, patients’ values, preferences, and situations in addition to evidence and are involved in health-related decision making together” [1]. The National Institute of Medicine has called for the implementation of SDM to improve the quality of patient-centered healthcare [2]. SDM is considered to be the ethical responsibility of physicians because supporting patients’ decision making leads to improved patient autonomy [3] and is widely recognized as the best practice in decision making, particularly with regard to preference-sensitive decisions [4].

Although prostate cancer treatment is beneficial, it carries various potential harms and side effects (urinary incontinence and sexual dysfunction after surgery or radiotherapy, side effects of hormone therapy or chemotherapy, psychological distress, etc.) depending on the nature of the treatment [5, 6]. How much risk patients want to avoid is decided based on their beliefs, values, and the treatment’s potential impact on their daily life, and they are required to weigh up the benefits of receiving treatment against the disadvantages associated with treatment [7]. However, Scherr et al. have shown that the only predictor of treatment received is the physician’s recommendation which, in turn, is based on Gleason score and age [8]. Many patients with prostate cancer feel that they are not provided with enough information when making a decision about which treatment to choose [9, 10], and the patients who feel they were not provided with enough information tend to regret their decision [11]. Meanwhile, those patients with prostate cancer who are more actively involved in decision making have improved knowledge of the disease and experience less decisional conflict and regret in their decision making process [12]. It is also known that improvement in patient satisfaction with the decision making process contributes to patients’ long-term quality of life (QOL) post-treatment [13]. SDM helps patients with prostate cancer feel as though they have been provided with sufficient information, as well as increasing their knowledge [14]. Thus, SDM constitutes an intervention to promote active involvement in treatment-oriented decision making for patients with prostate cancer and should be introduced into daily clinical practice as an intervention that may positively impact QOL.

Among the ever-increasing number of patients with prostate cancer, 80 to 90% have androgen-dependent tumors that shrink in the absence of androgen [15]. Therefore, in addition to surgery, radiotherapy, and chemotherapy, hormone therapy is one option for some types of prostate cancer [15, 16]. Hormone therapy has been used to treat patients with prostate cancer as many of them are elderly, and prefer such conservative treatment over surgery and radiation therapy when choosing a treatment option [17]. New hormonal agents have been developed in recent years, and some have been approved in Japan in the last 5 to 6 years. While various hormonal agents have similar mode of actions and adverse events, costs and therapeutic efficacies are not identical. In clinical practice, therefore, it is sometimes difficult for physicians to determine which drug should be used. Physicians must recommend evidence-based therapies when making a treatment decision together with patients. However, patients may experience severe adverse events. Therefore, it is important for physicians to communicate closely with patients choosing the best therapy for patients. However, for hormone therapy for prostate cancer, there are no reports that describe the use of the reliable and valid nine-item Shared Decision Making Questionnaire (SDM-Q-9) or the Shared Decision Making Questionnaire for Doctors (SDM-Q-Doc) surveys, nor are there any reports on the provision of information to patients at the start of hormone therapy, the current status of SDM, or any investigation of the relationship between SDM and treatment satisfaction. Thus, it is not clear which factors are associated with the relationship between the implementation of SDM and treatment satisfaction.

Although SDM should play an important role in a decision making about treatment including hormone therapy because it is complex and highly preference-sensitive, its association with patients’ treatment satisfaction is not fully understood. If SDM and patients’ treatment satisfaction were associated, we would be able to demonstrate that SDM is important for prostate cancer patients when a decision is made. The aim of this study was to clarify whether SDM was associated with the physicians’ explanations and perceived effective decision making for patients, and thus with the treatment satisfaction. In this study, we tested Hypothesis 1: “Patients’ perception of SDM is associated with treatment satisfaction, mediated by satisfaction with physicians’ explanations and perceived effective decision making” and Hypothesis 2: “The amount of information provided to patients by physicians on diseases and treatment is associated with treatment satisfaction mediated by patients’ perceived SDM and satisfaction with physicians’ explanations” in the field of hormone therapy for prostate cancer.

## Methods

### Study design and participants

This study was designed to be cross-sectional and was conducted using an online panel via a private research company in Japan, in which data were planned to be collected from at least 100 patients and 150 physicians. To collect the data, the private research company sent an email message with a URL link to the questionnaire (see Additional file 1) to 433 cancer patients among the panel registrants, requesting participation in the survey. Among all patients, the study included those who had received or were receiving hormone therapy for prostate cancer and responded to all items. We obtained online answers from 124 respondents (the “patient group”). The private research company manages panel registrants who respond to surveys at least once a year as active members. They have been developing their own panels without any external funds.

Of the panel registrants, the study included physicians who are urology specialists and have started drug therapy for prostate cancer in the past year. We approached 7251 urologists, asking those who had treated more than 10 prostate cancer patients in the past month and had at least 3 years of clinical experience to respond to the questionnaire (see Additional file 2) by email, obtaining online answers from 150 physicians (the “physician group”).

### Measures

#### Perceived shared decision making

The Japanese version of the SDM-Q-9, which consists of nine items, was used to evaluate the SDM process from perspective of patients [18], and that of the SDM-Q-Doc, which also consists of nine items, was used to measure the extent to which physicians perceive themselves to have conducted SDM with their patients [19].

These two instruments were developed in Germany and have been translated into English, French, Spanish, Portuguese, and other languages, including Japanese [20]. Response options were on a six-point scale from 1 (completely disagree) to 6 (completely agree) and the points were converted to a total out of 100 with higher scores indicating higher satisfaction in the SDM process.

#### Levels of satisfaction with physicians’ explanations and treatment satisfaction

Patients were asked “How satisfied were you with the explanation provided by the doctor when you started drug therapy for prostate cancer for the first time?” to gauge their satisfaction with physicians’ explanations, and “How satisfied were you with the first session of drug therapy?” to gauge their treatment satisfaction. For the answer choices, we used a five-point Likert scale from 1 (strongly disagree) to 5 (strongly agree).

We asked physicians “How satisfied were the patients with your explanation?” to gauge the level of patients’ satisfaction with physicians’ explanations, and “How satisfied were the patients with their first drug therapy?” to gauge patients’ level of treatment satisfaction. For answer options, we used a six-point scale from 1 (I think 0% of patients and their families are satisfied) to 6 (I think 100% of patients and their families are satisfied).

#### Perceived effective decision making

We asked the patient group about their effective decision making. The level of effective decision making refers to how satisfied the patients are with their decision making after obtaining information, making a decision consistent with their own values, and having no uncertainty about their decision. We used the subscale “Perceived effective decision making” from the Japanese version of the decisional conflict scale [21, 22]. This scale comprises of four items, such as “I feel I have made an informed choice.” We obtained answers using a five-point scale from 1 (strongly disagree) to 5 (strongly agree) with their decision making. The points were converted to a total out of 100 [23].

We asked the physician group about their perceptions of patients’ effective decision making. We modified the four items, such as “I feel I have made an informed choice.” to “Your patients feel they have made an informed choice.” We obtained the answers using a five-point scale from 1 (strongly disagree) to 5 (strongly agree). Higher scores indicated that patients had a higher level of effective decision making.

#### Amount of information on disease and care provided by physicians at the start of drug therapy

We asked the patient group whether their physicians or other healthcare workers explained 20 items to them, including diagnostic results, prostate cancer, the treatment to be started, its impact on daily life, and support systems, when drug therapy was started for the first time. Our study members created these 20 items as information that physicians should provide to patients from the viewpoint of physicians specialized in prostate cancer and representatives of prostate cancer patients’ advocacy group. We analyzed the number of items with physicians’ explanations to gauge the amount of information provided to patients.

We asked the physician group whether they explained 20 items, including diagnostic results, prostate cancer as a disease, the treatment to be started, introduction of support system, and impact on daily life when drug therapy was started for the first time, to all patients and families. We also asked whether they only partially explained these items, and whether other healthcare workers provided an explanation. We used the number of items that the physicians explained to patients by themselves in order to analyze the amount of information provided to patients.

#### Demographic and clinical variables

The patient group was asked about their age, occupation, years after diagnosis, years after drug therapy, the presence/absence of metastasis, and previous treatment. The physician group was asked about their age, sex, place of work, number of prostate cancer patients in the preceding month, number of patients who started initial drug therapy in the preceding year and years of experience.

### Statistical analysis

To evaluate the reliability and validity of SDM-Q-9 and SDM-Q-Doc, Cronbach’s alpha was calculated to examine internal consistency of the items. For construct validity, confirmatory factor analysis (CFA) was conducted, under an assumption that the questionnaire had a unifactorial structure. In CFA, the Comparative Fit Index (CFI) and the root mean square error of approximation (RMSEA) were used as the model fit indices. A CFI value of 0.90 or larger is generally considered to indicate acceptable model fit. An RMSEA value of less than 0.05 represents good fit, and a value < 0.08 is acceptable [24].

We developed a path diagram and performed path analysis to confirm Hypotheses 1 and 2, or the relationship between SDM, the amount of information provided by physicians to patients, perceived effective decision making, physicians’ explanations, and the level of treatment satisfaction. CFA was performed to examine fitness of the path diagram. Statistical significance of path coefficients was tested to see if a similar relationship was observed between the patient and physician groups. SPSS and Amos ver. 25.0 were used as statistical software.

## Results

### Participant characteristics

The data were collected between January 25 and February 2 in 2018. Table 1 shows the characteristics of the patient group. The largest number of patients were in their 70s, with a mean age of 71.5 (standard deviation [SD] ± 7.6) and median age of 75. Among the participants, 62.9% had no occupation. The largest number of patients (23.4% of all participants) had been diagnosed with prostate cancer for < 2 years, and the largest number of patients (75.8%) had no metastasis at diagnosis. Regarding patients’ prior treatment, 100.0% received hormone therapy (since the study was conducted on patients who had previously been treated with hormone therapy), followed by 38.7% who had received external radiation therapy and 32.3% who had undergone surgery.

**Table 1:** Characteristics of study participants: patient group (*n* = 124)

Variables	n	%
Age group		
50–59	7	5.6
60–69	40	32.3
70–79	57	46.0
80–89	20	16.1
Age (mean ± SD)	71.5	7.6
Occupation		
Employed (full-time)	22	17.7
Employed (part-time)	19	15.3
Homemaker	1	0.8
Unemployed	78	62.9
Other	4	3.2
Years after diagnosis		
< 2 years	29	23.4
2–4 years	24	19.4
4–6 years	28	22.6
6–10 years	24	19.4
> 10 years	19	15.3
Years after drug therapy		
< 2 years	46	37.1
2–4 years	21	16.9
4–6 years	27	21.8
6–10 years	17	13.7
> 10 years	13	10.5
Metastasis at diagnosis		
Bone metastasis	18	14.5
Lymph node metastasis	13	10.5
Organ metastasis (the lung, liver, etc.)	4	3.2
No metastasis	94	75.8
Unknown	3	2.4
Previous treatment		
Surgical therapy	40	32.3
External irradiation	48	38.7
Brachytherapy	3	2.4
Hormone drugs (injection, oral drugs, etc.)	124	100.0
Chemotherapy (anticancer drugs)	14	11.3
Bone-modifying agents (drugs that suppress metastatic bone lesions)	7	5.6
Radiopharmaceuticals (radium, strontium)	1	0.8

Table 2 shows the characteristics of the physician group. Most physicians were male (98.7%) and the majority were in their 40s, with a mean age of 45.1 (SD: ± 8.2) and median age of 45. The largest number of physicians worked for public general hospitals (40.0%), followed by private general hospitals and university hospitals. An equal number of physicians (50 physicians, 33.3% each) examined 39, 40–69, and > 70 patients with prostate cancer in the past month. Regarding the number of patients who started their first drug therapy in the previous year, 30.0% of physicians answered 10–19 patients and 42.0% answered > 20 patients.

**Table 2:** Characteristics of study participants: physician group (*n* = 150)

Variables	n	%
Gender		
Men	148	98.7
Women	2	1.3
Age group		
≤ 39	36	24.0
40–49	77	51.3
> 50	37	24.7
Age (mean ± SD)	45.1	8.2
Place of work		
General hospital (public)	60	40.0
General hospital (private)	56	37.3
University hospital	34	22.7
Number of patients with prostate cancer in the past month		
≤ 39	50	33.3
40–69	50	33.3
> 70	50	33.3
Number of patients who started initial drug therapy in the past year		
≤ 9	42	28.0
10–19	45	30.0
> 20	63	42.0

### Relationship between SDM and perceived effective decision making, satisfaction with physicians’ explanations, and treatment satisfaction

**Fig. 1:**
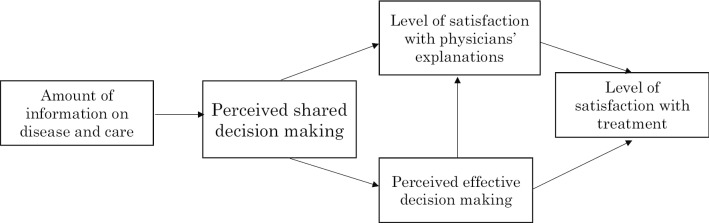
Conceptual framework for path analyses. Path diagram was developed to evaluate a relationship between perceived shared decision making, physicians’ explanations, and treatment satisfaction in patients with prostate cancer receiving hormone therapy. Squares represent measured variables

To test Hypotheses 1 and 2, we prepared a path diagram for the patient group and examined the relationship between perceived SDM, the amount of information provided by physicians, perceived effective decision making, satisfaction with physicians’ explanations, and treatment satisfaction (Fig. 1). Additionally, we used the same diagram for the physician group to confirm whether the perceptions of the physician group were the same as those of the patient group.

**Fig. 2:**
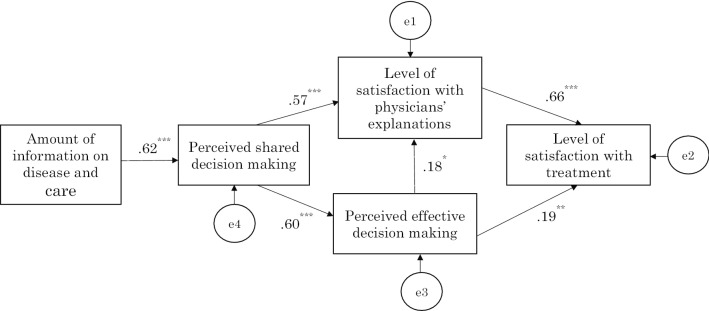
Path diagram for the patient group. Significant path coefficient was obtained for all cases. Squares represent measured variables, and values are path coefficients. e1 – e4 represent errors. *p < 0.05, **p < 0.01, ***p < 0.001

The fitness level of the patient group path diagram was CFI = 1.00 with RMSEA < 0.001, and all path coefficients were significant (*P* < 0.001; the path coefficients from perceived effective decision making to satisfaction with physicians’ explanations and treatment satisfaction were *P* = 0.028 and *P* = 0.004, respectively) (Fig. 2).

**Fig. 3:**
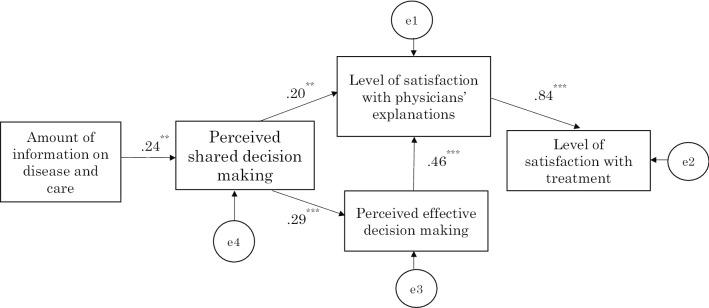
Path diagram for the physician group. Significant path coefficient was obtained for all cases except a path from perceived effective decision making to treatment satisfaction (path coefficient 0.07, *P* = 0.15). Squares represent measured variables, and values are path coefficients. e1 – e4 represent errors. **p < 0.01, ***p < 0.001

The fitness of the path diagram of the physician group was CFI = 0.989 and RMSEA = 0.076, and all path coefficients were significant (*P* < 0.001; the path coefficients from the amount of information to perceived SDM and perceived SDM to satisfaction with physicians’ explanations were *P* = 0.003 and *P* = 0.006, respectively) (Fig. 3). In the path diagrams of the patient group and physician group, the significant paths were almost the same except for the path from perceived effective decision making to treatment satisfaction.

The path coefficient from satisfaction with the physicians’ explanations to treatment satisfaction was the largest (0.66 in patients and 0.84 in physicians), indicating a strong relationship. Furthermore, SDM was indirectly related through perceived effective decision making, in addition to the path directly associated with satisfaction with physicians’ explanations, which supported Hypothesis 1.

In both groups, more SDM was implemented when more information was provided about the disease and care at the start of the first drug therapy. The amount of information relayed did not directly relate to satisfaction with physicians’ explanations – when a path is drawn from the amount of information to satisfaction with physicians’ explanations, the path coefficients in patients and physicians were − 0.03, *P* = 0.70 and 0.11, *P* = 0.13, respectively – and was mediated by SDM, which supported Hypothesis 2.

However, even the significant paths for both patient and physician groups had path coefficients of different magnitudes. The path coefficient for the path from the amount of information about the disease and care provided by physicians to SDM was 0.62 in the patient group, while it was 0.24 in the physician group. The path coefficient from SDM to satisfaction with physicians’ explanations was 0.57 in the patient group and 0.20 in the physician group. Similarly, the path coefficient from perceived SDM to perceived effective decision making was 0.60 in the patient group and 0.29 in the physician group.

The path coefficient from perceived effective decision making to satisfaction with physicians’ explanations was 0.18 in the patient group and 0.46 in the physician group. From perceived effective decision making to treatment satisfaction, it was only possible to draw a significant path for the patient group with a path coefficient of 0.19, but not for the physician group (the path coefficient obtained by drawing such a path was 0.07 and *P* = 0.15).

### Reliability and validity of SDM-Q-9 and SDM-Q-Doc

To test the reliability of SDM-Q-9 and SDM-Q-Doc, Cronbach’s alpha coefficient was calculated. Cronbach’s alpha coefficients of the SDM-Q-9 for the patient group and the SDM-Q-Doc for the physician group were 0.947 and 0.919, respectively. In both cases, the coefficient did not increase on deleting any items, and the highest value was shown when using all nine items.

The validity of SDM-Q-9 and SDM-Q-Doc was confirmed using confirmatory factor analysis. In the SDM-Q-9 survey for the patient group, the CFI was 0.974 and the RMSEA was 0.096. Some error covariances were observed between items with similar question texts (items 1 and 2; item 3 with 4 and 6; items 7 and 8; and items 8 and 9); factor loadings in the confirmatory factor analysis (CFA loadings) were > 70 in all cases, and unifactorial structure was confirmed. In the SDM-Q-Doc used for the physician group, the CFI was 0.970 and the RMSEA was 0.090. As with the patient group, some error covariances were observed in the physician group between items with similar question texts (items 1 and 2; items 3 and 4; items 4 and 8; items 7 and 8; and items 8 and 9), but the factor loadings (the CFA loadings) were > 0.60 in all cases, and unidimensional structure was confirmed.

## Discussion

The results of the questionnaire for this patient group show that the amount of information provided by physicians before starting hormone therapy strongly influenced SDM. However, the amount of information provided by physicians did not significantly influence the level of satisfaction with physicians’ explanations but did influence the level of satisfaction with the physicians’ explanations mediated by SDM. Nejati et al. discussed the validity of SDM-Q-9 and SDM-Q-Doc in the oncology setting [25]. SDM-Q-9 was significantly positively correlated with satisfaction with physicians (in terms of information provided by physicians, risk of recurrence, side effects of treatment, time spent on providing information to patients) and patients’ satisfaction. The results of this study mirror those of Nejati et al. Orom et al. [26] reported that patients who were more actively involved in decision making were more satisfied with the decision but had greater difficulty coming to a decision. To improve the level of satisfaction with physicians’ explanations, it is not necessarily enough to just increase the amount of information. Communication that provides the highest level of assistance to patients in their decision making appears to be a vital factor in the SDM process. In this patient group, the level of effective decision making also influenced the level of satisfaction with physicians’ explanations, which was less influential than SDM. These results indicate that the decision making process is more important for patients than the results of the decision reached in communication with physicians at the time of treatment decision making. It should also be noted that no direct relationship was observed between SDM and treatment satisfaction. SDM influenced treatment satisfaction mediated by satisfaction with both physicians’ explanations and decision making. The degree of influence on treatment satisfaction was greater for the level of satisfaction with physicians’ explanations than with the level of effective decision making. The results from the patient group in this study suggest that when physicians used SDM to help patients actively engage in treatment decision making while presenting a wide range of information (on prostate cancer, treatment options, their benefits and risks, their impact on life, and support, etc.), the levels of effective decision making and with physicians’ explanations improved and, accordingly, the level of patients’ treatment satisfaction improved.

The term “medical paternalism” has been used in Japan for many years, and it has been asserted that many patients want paternalistic medical care. However, recently, an increasing number of patients have exhibited the desire to be more actively involved in decision making. Approximately 40% of patients with prostate cancer want to be actively involved in decision making, almost 50% of patients want to decide with physicians, and just 10% of patients prefer paternalistic decision making [27]. The results of this study also indicated that SDM is highly correlated with patients’ decision making and treatment satisfaction, a finding similar to that of Schaede et al. In Japan, healthcare professionals such as physicians need to alter their perception that many patients want to take a passive role in selecting treatment, and ought to consider strategies for introducing SDM into the clinical setting.

According to a report on the investigation of patients with colorectal and lung cancers conducted by Kehl et al. [28], when decision making was controlled by physicians, patients assessed the overall quality of care and the quality of physicians’ communication as low, a finding that was independent of patients’ preferences regarding their decision. Therefore, healthcare professionals such as physicians should introduce SDM so that all patients can be involved in treatment decision making.

Based on the results of the questionnaire survey of the physician group, we constructed a path diagram similar to that of the patient group. However, the magnitude of the relationship between the amount of information provided by physicians and SDM, SDM and the level of effective decision making and the proportion of patients satisfied with their explanation was smaller than that of the patient group. These results suggest that physicians, compared to patients, may underestimate the importance of SDM before starting hormone therapy. Driever et al. [29] reported that 31.0% made a decision using a paternalistic approach because they perceived that it was difficult for patients to make a decision, indicating that physicians and other healthcare professionals may create a barrier to SDM. To overcome such barrier, it is necessary to provide SDM communication skill training [30] and develop patient decision aids for Japanese patients, which are assistive tools that promote SDM.

To our knowledge, this is the first questionnaire survey of both Japanese patients with prostate cancer and Japanese physicians using SDM-Q-9 and SDM-Q-Doc. The reliability and validity of both instruments were confirmed before we tested the hypotheses using statistical analysis. As a result, Cronbach’s alpha coefficient was > 0.9 in both Japanese versions of the SDM-Q-9 and SDM-Q-Doc surveys, supporting the internal consistency reliability. Cronbach’s alpha coefficient of the original German version of SDM-Q-9 was 0.938 [31] and SDM-Q-Doc was 0.88 [32], indicating that the reliability of Japanese versions is equivalent to the original versions. The CFA results meet CFI criteria and, therefore, the reliability and validity of the Japanese versions of the SDM-Q-9 and SDM-Q-Doc surveys were confirmed in the field of prostate cancer. Taken together, these findings indicate that both instruments can be used in researching the field of prostate cancer in Japan.

The limitations of this study were as follows: the subjects were panel members registered with an Internet survey company and the correspondence of the patient group and the physician group was not considered. Although, on average, patients had received hormone therapy 5 years prior to the survey, some patients needed to recall hormone therapy that took place over 5 years prior to the survey. Therefore, the data may not be accurate for some patients due to the memory-dependent nature of the data. In addition, prostate cancer hormone therapy ranges from adjuvant and combination therapy for localized prostate cancer to treatment of distant metastases [33]. Therefore, the patient group in this study also included 28.2% of patients with advanced cancer in whom metastases had been diagnosed, which may have affected the survey results. In the survey of the physician group, physicians’ answers to the question about their patients’ level of satisfaction were based on the perception of physicians. In real clinical settings, physicians determine their patients’ satisfaction with a patient satisfaction survey conducted at many hospitals or based on conversations with their patients during hospital visits. However, in our study, we did not ask physicians to indicate how they determined patients’ satisfaction levels. Therefore, physicians’ answers may be biased. In the future, it is expected that the actual status quo will be better reflected by conducting a survey in which patients with prostate cancer are surveyed at the time of treatment decision making and the physicians who treat them complete a corresponding survey in which the treatment options themselves and the stage of patients’ prostate cancer are considered. This study is also limited because it utilized the cross-sectional data that did not allow a firm conclusion to be drawn about mediation effects between the investigated variables. In addition, the amount of information provided to patients and SDM measured in this study were perceived by physicians and patients. Therefore, they do not represent subjective measures of whether SDM was actually performed or physicians actually provided such information. In addition, reliability and validity of measures (the amount of information provided to patients, and levels of satisfaction with the amount of information provided to patients and treatment satisfaction) were not fully confirmed. In a future study, a relationship between these measures and SDM needs to be studied with less bias.

As mentioned above, this study has some limitations. However, there have been no studies in which SDM was measured using SDM-Q-9 and SDM-Q-Doc in an oncology setting in Japan. The reliability and validity of the Japanese versions of the SDM-Q-9 and SDM-Q-Doc surveys have been confirmed, and we examined the relationship between SDM, the amount of information provided by physicians, the level of effective decision making and physicians’ explanations, and patients’ treatment satisfaction using path analysis and compared the results with those of physicians, all of which are unique features of this study. Prior to this, to our knowledge, there have been no similar published reports. Jung et al. [34] reported that better information on side effects in patients with prostate cancer receiving endocrine therapy was associated with better medication adherence. SDM (including discussion of the side effects of endocrine therapy, its impact on life and measures for it, and support systems) provided by physicians may also improve patients’ adherence to medication. Moreover, SDM in an oncology setting is negatively correlated with depression and anxiety [25], and it has been shown that treatment satisfaction affects QOL [13].

## Conclusions

In this study, the perceived SDM by patients with prostate cancer at the start of hormone therapy increased when physicians provided more information, and this influenced treatment satisfaction mediated by satisfaction with both physicians’ explanations and perceived effective decision making. Path analysis revealed a particularly strong relationship between SDM, satisfaction with physicians’ explanations, and treatment satisfaction. Physicians who were involved in the treatment of patients with prostate cancer also showed similar results to those of patients, but compared with patients, they may tend to underestimate the importance of SDM before starting hormone therapy. We therefore suggest that physicians communicate using SDM to help patients become actively involved in decision making about treatment, while presenting them with a wide range of information; effective decision making and physicians’ explanations may be improved, thereby improving patients’ treatment satisfaction.

## Supplementary Information

**Additional file 1.** Questionnaire for patients.

**Additional file 2.** Questionnaire for physicians.

## Data Availability

Requests for the study materials and dataset used to support the conclusions of this article should be directed to the corresponding author.

## Abbreviations

CFA: Confirmatory factor analysis
CFI: Comparative fit index
QOL: Quality of Life
RMSEA: Root mean square error of approximation
SD: Standard deviation
SDM: Shared decision making
SDM-Q-9: Nine-item shared decision making questionnaire
SDM-Q-Doc: Shared decision making questionnaire for doctors
