# Supplementary material for: Shared decision making, physicians’ explanations, and treatment satisfaction: a cross-sectional survey of prostate cancer patients
Source: BMC Med Inform Decis Mak. 2020 Dec 14;20:334. doi: 10.1186/s12911-020-01355-z (PMC7734751; doi:10.1186/s12911-020-01355-z)
Supplement: Supplementary file 1 — Additional file 1. Questionnaire for patients. [file 12911_2020_1355_MOESM1_ESM.docx]

Questionnaire for patients

- Please indicate your age

( ) years old

- Please indicate your gender

1 Male

2 Female

- Please answer all that apply from the following about prostate cancer treatment you have ever received at medical institutions.

|  | | All treatments you have ever received |
| --- | --- | --- |
| 1 | Surgical therapy | □ |
| 2 | External radiotherapy | □ |
| 3 | Brachytherapy | □ |
| 4 | Hormone preparations (e.g., injections and oral drugs) | □ |
| 5 | Chemotherapy [anticancer agents] | □ |
| 6 | Bone modifying agents (drugs to suppress metastatic bone lesions) | □ |
| 7 | Radiopharmaceuticals (radium, strontium) | □ |
| 8 | Other ( ) | □ |

- When were you diagnosed with prostate cancer? If you do not remember the detailed time, please answer an approximate time.

Time when diagnosed with prostate cancer ( ) Year (Christian Era) ( ) Month

- When did you start drug therapy [injections, oral drugs, hormone therapy or chemotherapy (anticancer agents)]? If you do not remember the detailed time, please answer an approximate time.

Time when you started drug therapy [injections, oral drugs, hormone therapy or chemotherapy (anticancer agents)] ( ) Year (Christian Era) ( ) Month

- What was the metastatic status at diagnosis of prostate cancer?

1 Bone metastases

2 Lymph node metastases

3 Visceral metastases (e.g., lungs, liver)

4 No metastasis

5 I don’t know/I don’t remember

- Please choose only one that applies to your working status

1 Full-time worker (≥ 35 hours a week)

2 Part-time worker (< 35 hours a week)

3 Full-time homemaker

4 Student

5 Unemployed

6 Other ( )

- How did the physician or healthcare professional other than physician (e.g., pharmacist/nurse) explain when you first started drug therapy (hormone therapy) for prostate cancer?

Please choose all that apply from the followings.

|  | | a | b |
| --- | --- | --- | --- |
|  | Diagnostic results | Explained by the physician | Explained by a healthcare professional other than the physician |
| 1 | Extension of cancer [stage/degree of progression (early, advanced)] | □ | □ |
| 2 | Presence of metastasis | □ | □ |
| 3 | PSA level | □ | □ |
| 4 | GS (Gleason score)/malignancy of cancer | □ | □ |
| 5 | Other diagnostic results ( ) | □ | □ |
| 6 | Neither was explained | □ | □ |

- How did the physician or healthcare professional other than physician (e.g., pharmacist/nurse) explain when you first started drug therapy (hormone therapy) for prostate cancer?

Please choose all that apply from the followings.

|  | | a | b |
| --- | --- | --- | --- |
|  | Prostate cancer as a disease | Explained by the physician | Explained by a healthcare professional other than the physician |
| 1 | General explanation about prostate cancer | □ | □ |
| 2 | Chance of recurrence | □ | □ |
| 3 | Prognosis (future prospect of the disease) | □ | □ |
| 4 | Possible pain, fracture and/or paralysis caused by bone metastasis | □ | □ |
| 5 | Other things about prostate cancer as a disease ( ) | □ | □ |
| 6 | Neither was explained | □ | □ |

- How did the physician or healthcare professional other than physician (e.g., pharmacist/nurse) explain when you first started drug therapy (hormone therapy) for prostate cancer?

Please choose all that apply from the followings.

|  | | a | b |
| --- | --- | --- | --- |
|  | Treatment to be started | Explained by the physician | Explained by a healthcare professional other than the physician |
| 1 | Presence of treatment options other than the treatment conducted | □ | □ |
| 2 | Data on treatment and drugs | □ | □ |
| 3 | Expected therapeutic effects | □ | □ |
| 4 | Expected adverse reactions | □ | □ |
| 5 | Duration of treatment or drug administration | □ | □ |
| 6 | Treatment cost | □ | □ |
| 7 | Other things about the treatment to be started ( ) | □ | □ |
| 8 | Neither was explained | □ | □ |

- How did the physician or healthcare professional other than physician (e.g., pharmacist/nurse) explain when you first started drug therapy (hormone therapy) for prostate cancer?

Please choose all that apply from the followings.

|  | | a | b |
| --- | --- | --- | --- |
|  | Introduction of support system | Explained by the physician | Explained by a healthcare professional other than the physician |
| 1 | High-cost medical care benefit system | □ | □ |
| 2 | Care for pain and physical unpleasant symptoms | □ | □ |
| 3 | Care for mental issues including distress | □ | □ |
| 4 | Cancer consultation and support center, patients association, etc. | □ | □ |
| 5 | Introduction of other support systems ( ) | □ | □ |
| 6 | Neither was explained | □ | □ |

- How did the physician or healthcare professional other than physician (e.g., pharmacist/nurse) explain when you first started drug therapy (hormone therapy) for prostate cancer?

Please choose all that apply from the followings.

|  | | a | b |
| --- | --- | --- | --- |
|  | Impact on daily life | Explained by the physician | Explained by a healthcare professional other than the physician |
| 1 | Impact on daily life including work and hobbies | □ | □ |
| 2 | Possible impact on sex life | □ | □ |
| 3 | Other impact on daily life ( ) | □ | □ |
| 4 | Neither was explained | □ | □ |

- How satisfied were you about the explanation from the physician when you or your family member first started drug therapy (hormone therapy) for prostate cancer?

How satisfied were you about the treatment?

Please choose only one that best describes in each item.

|  | | Very satisfied | Somewhat satisfied | Neither satisfied nor dissatisfied | Somewhat dissatisfied | Very dissatisfied |
| --- | --- | --- | --- | --- | --- | --- |
| 1 | Level of satisfaction with doctors’ explanation | 1 | 2 | 3 | 4 | 5 |
| 2 | Level of satisfaction with treatment | 1 | 2 | 3 | 4 | 5 |

- These are the questions about the drug (hormone therapy) you started most recently among the drugs you have been taking.

Please choose the best describes in each of the following items.

|  | | Completely disagree | Strongly disagree | Some-what disagree | Some-what agree | Strongly agree | Completely agree |
| --- | --- | --- | --- | --- | --- | --- | --- |
| 1 | My doctor made clear that a decision needs to be made | 1 | 2 | 3 | 4 | 5 | 6 |
| 2 | My doctor wanted to know exactly how I want to be involved in making the decision | 1 | 2 | 3 | 4 | 5 | 6 |
| 3 | My doctor told me that there are different options for treating my medical condition | 1 | 2 | 3 | 4 | 5 | 6 |
| 4 | My doctor precisely explained the advantages and disadvantages of the treatment options | 1 | 2 | 3 | 4 | 5 | 6 |
| 5 | My doctor helped me understand all the information | 1 | 2 | 3 | 4 | 5 | 6 |
| 6 | My doctor asked me which treatment option I prefer | 1 | 2 | 3 | 4 | 5 | 6 |
| 7 | My doctor and I thoroughly weighed the different treatment options | 1 | 2 | 3 | 4 | 5 | 6 |
| 8 | My doctor and I selected a treatment option together | 1 | 2 | 3 | 4 | 5 | 6 |
| 9 | My doctor and I reached an agreement on how to proceed | 1 | 2 | 3 | 4 | 5 | 6 |

- Please recall when you started the drug (hormone therapy) most recently among the drugs you have been taking.

Please choose the best describes in each of the following items.

|  | | Strongly agree | Agree | Neither agree nor disagree | Disagree | Strongly disagree |
| --- | --- | --- | --- | --- | --- | --- |
| 1 | I feel I have made an informed choice | 1 | 2 | 3 | 4 | 5 |
| 2 | My decision shows what is important to me | 1 | 2 | 3 | 4 | 5 |
| 3 | I expect to stick with my decision | 1 | 2 | 3 | 4 | 5 |
| 4 | I am satisfied with my decision | 1 | 2 | 3 | 4 | 5 |
