# Supplementary material for: Shared decision making, physicians’ explanations, and treatment satisfaction: a cross-sectional survey of prostate cancer patients
Source: BMC Med Inform Decis Mak. 2020 Dec 14;20:334. doi: 10.1186/s12911-020-01355-z (PMC7734751; doi:10.1186/s12911-020-01355-z)
Supplement: Supplementary file 2 — Additional file 2. Questionnaire for physicians. [file 12911_2020_1355_MOESM2_ESM.docx]

Questionnaire for physicians

- Please indicate your gender

1 Male

2 Female

- Please indicate your age

( ) years old

- Please indicate the years of your clinical experience after you completed your residency.

( ) Year

- Please indicate the type of the medical institution of your main work place.

1 General hospital (private)

2 General hospital (public)

3 University hospital

4 Clinic

5 Other ( )

- Please indicate the number of patients you have consulted in the last month.

1 Entire patients ( ) patients/month

2 Of them, patients with prostate cancer ( ) patients/month

- Please indicate the number of patients you have consulted who started initial drug therapy (excluding adjuvant/neoadjuvant therapies) in the last year.

Patients who started the initial drug therapy in the last year

(excluding adjuvant/neoadjuvant therapies) ( ) patients/year

- How do you or healthcare professionals other than physician (e.g., pharmacist/nurse) explain when your patient first start drug therapy for prostate cancer?

Please choose all that apply from the followings.

|  | | a | b | c |
| --- | --- | --- | --- | --- |
|  | Diagnostic results | You explain to all your patients and their family members | You explain to some of your patients and their family members | Healthcare professionals other than physician explain |
| 1 | Extension of cancer [stage/degree of progression (early, advanced)] | □ | □ | □ |
| 2 | Presence of metastasis | □ | □ | □ |
| 3 | PSA level | □ | □ | □ |
| 4 | GS (Gleason score)/malignancy of cancer | □ | □ | □ |
| 5 | Other diagnostic results ( ) | □ | □ | □ |
| 6 | I do not explain any of the above | □ | □ | □ |

- How do you or healthcare professionals other than physician (e.g., pharmacist/nurse) explain when your patient first start drug therapy for prostate cancer?

Please choose all that apply from the followings.

|  | | a | b | c |
| --- | --- | --- | --- | --- |
|  | Prostate cancer as a disease | You explain to all your patients and their family members | You explain to some of your patients and their family members | Healthcare professionals other than physician explain |
| 1 | General explanation about prostate cancer | □ | □ | □ |
| 2 | Chance of recurrence | □ | □ | □ |
| 3 | Prognosis (future prospect of the disease) | □ | □ | □ |
| 4 | Possible pain, fracture and/or paralysis caused by bone metastasis | □ | □ | □ |
| 5 | Other things about prostate cancer as a disease ( ) | □ | □ | □ |
| 6 | I do not explain any of the above | □ | □ | □ |

- How do you or healthcare professionals other than physician (e.g., pharmacist/nurse) explain when your patient first start drug therapy for prostate cancer?

Please choose all that apply from the followings.

|  |  | a | b | c |
| --- | --- | --- | --- | --- |
|  | Treatment to be started | You explain to all your patients and their family members | You explain to some of your patients and their family members | Healthcare professionals other than physician explain |
| 1 | Presence of treatment options other than the treatment conducted | □ | □ | □ |
| 2 | Data on treatment and drugs | □ | □ | □ |
| 3 | Expected therapeutic effects | □ | □ | □ |
| 4 | Expected adverse reactions | □ | □ | □ |
| 5 | Duration of treatment or drug administration | □ | □ | □ |
| 6 | Treatment cost | □ | □ | □ |
| 7 | Other things about the treatment to be started ( ) | □ | □ | □ |
| 8 | I do not explain any of the above | □ | □ | □ |

- How do you or healthcare professionals other than physician (e.g., pharmacist/nurse) explain when your patient first start drug therapy for prostate cancer?

Please choose all that apply from the followings.

|  |  | a | b | c |
| --- | --- | --- | --- | --- |
|  | Introduction of support system | You explain to all your patients and their family members | You explain to some of your patients and their family members | Healthcare professionals other than physician explain |
| 1 | High-cost medical care benefit system | □ | □ | □ |
| 2 | Care for pain and physical unpleasant symptoms | □ | □ | □ |
| 3 | Care for mental issues including distress | □ | □ | □ |
| 4 | Cancer consultation and support center, patients association, etc. | □ | □ | □ |
| 5 | Introduction of other support systems ( ) | □ | □ | □ |
| 6 | I do not explain any of the above | □ | □ | □ |

- How do you or healthcare professionals other than physician (e.g., pharmacist/nurse) explain when your patient first start drug therapy for prostate cancer?

Please choose all that apply from the followings.

|  |  | a | b | c |
| --- | --- | --- | --- | --- |
|  | Impact on daily life | You explain to all your patients and their family members | You explain to some of your patients and their family members | Healthcare professionals other than physician explain |
| 1 | Impact on daily life including work and hobbies | □ | □ | □ |
| 2 | Possible impact on sex life | □ | □ | □ |
| 3 | Other impact on daily life ( ) | □ | □ | □ |
| 4 | I do not explain any of the above | □ | □ | □ |

- How satisfied were your patients or their family members about your explanation when they first started drug therapy for prostate cancer?

How satisfied were your patients or their family members about the first drug therapy?

Please choose only one that best describes in each item.

|  |  | I think 100% of patients and their families are satisfied | I think 80% of patients and their families are satisfied | I think 60% of patients and their families are satisfied | I think 40% of patients and their families are satisfied | I think 20% of patients and their families are satisfied | I think 0% of patients and their families are satisfied |
| --- | --- | --- | --- | --- | --- | --- | --- |
| 1 | Level of satisfaction with doctors’ explanations | 1 | 2 | 3 | 4 | 5 | 6 |
| 2 | Level of satisfaction with treatment | 1 | 2 | 3 | 4 | 5 | 6 |

- These are the questions about overall treatment for prostate center and how you communicate with your patients about treatment.

Please choose the best describes in each of the following items.

|  |  | Completely disagree | Strongly disagree | Some-what disagree | Some-what agree | Strongly agree | Completely agree |
| --- | --- | --- | --- | --- | --- | --- | --- |
| 1 | I make clear to my patient that a decision needs to be made | 1 | 2 | 3 | 4 | 5 | 6 |
| 2 | I want to know exactly from my patient how he/she wants to be involved in making the decision | 1 | 2 | 3 | 4 | 5 | 6 |
| 3 | I tell my patient that there are different options for treating his/her medical condition | 1 | 2 | 3 | 4 | 5 | 6 |
| 4 | I precisely explain the advantages and disadvantages of the treatment options to my patient | 1 | 2 | 3 | 4 | 5 | 6 |
| 5 | I help my patient understand all the information | 1 | 2 | 3 | 4 | 5 | 6 |
| 6 | I asked my patient which treatment option he/she prefers | 1 | 2 | 3 | 4 | 5 | 6 |
| 7 | My patient and I thoroughly weighed the different treatment options | 1 | 2 | 3 | 4 | 5 | 6 |
| 8 | My patient and I selected a treatment option together | 1 | 2 | 3 | 4 | 5 | 6 |
| 9 | My patient and I reached an agreement on how to proceed | 1 | 2 | 3 | 4 | 5 | 6 |

- For your patients, please choose one that best describes in each of the following items regarding treatment option.

|  |  | Strongly agree | Agree | Neither agree nor disagree | Disagree | Strongly disagree |
| --- | --- | --- | --- | --- | --- | --- |
| 1 | Your patients feel they have made an informed choice | 1 | 2 | 3 | 4 | 5 |
| 2 | Your patients think their decision shows what is important to them | 1 | 2 | 3 | 4 | 5 |
| 3 | Your patients think they expect to stick with their decision | 1 | 2 | 3 | 4 | 5 |
| 4 | Your patients are satisfied with their decision | 1 | 2 | 3 | 4 | 5 |
